# One-year follow-up of epithelial corneal cell sheet allografts mounted on platelet poor plasma in rabbits

**Published:** 2009-12-15

**Authors:** Federico Luengo Gimeno, Victoria Lavigne, Silvia Gatto, J. Oscar Croxatto, Laura Correa, Juan E. Gallo

**Affiliations:** 1 Ophthalmology Department, Facultad de Ciencias Biomédicas, Universidad Austral, Pilar, Argentina; 2 Tissue engineering Department, Laboratorios Craveri, Buenos Aires, Argentina; 3 Transfusion Department, Hospital Universitario Austral, Universidad Austral, Pilar, Argentina; 4 Ocular Pathology Department, Fundación Oftalmológica Argentina "Jorge Malbran", Buenos Aires, Argentina

## Abstract

**Purpose:**

To evaluate the usefulness of epithelial corneal sheets mounted on platelet poor plasma (PPP) for allograft transplantation of rabbits with total limbal stem cell deficiency (LSCD) and to prove its efficacy at 1 year after surgery.

**Methods:**

LSCD was induced in 21 female rabbits by mechanical keratectomy. To configure the grafts, limbal biopsies were taken from male rabbits and cells were cultured on a fibroblast feeder layer grown on clotted autologous PPP. After keratectomy, grafts were sutured over the stroma. Control groups consisted of no implant or an implant of clotted PPP. Rabbits were euthanized at 3 and 12 months. Corneas and cultured sheets were processed for histopathology and immunohistochemistry (K3/12 and K19). Gender analysis was performed at 4 and 7 months.

**Results:**

One rabbit had endophthalmitis, and another died of no apparent cause. The rest of the animals treated had no inflammation, showed a stratified epithelium, keratin 3/12 expression, and no expression of keratin 19. At 1 year, seven of eight rabbits showed no LSCD or corneal rejection signs. Y chromosomes were detected at 4 and 7 months postoperatively. All controls showed LSCD signs, erratic epithelium, and minimal cell differentiation; they revealed a slight expression of K3/12 and an expression of K19 in patchy patterns.

**Conclusions:**

Allografts contributed to restoring a healthy eye surface without signs of graft rejection. This technique seems to be a promising procedure for bilateral ocular surface diseases and may be useful for new therapeutic strategies.

## Introduction

Limbal stem cell deficiency (LSCD) can be presented as recurrent and persistent epithelial defects with surface irregularities [1]. This situation develops in secondary infection and/or long-term inflammation, leading to secondary corneal opacification and neovascularization. The most severe sequelae to LSCD is corneal conjunctivalization, which is believed to be the consequence of the inability of corneal epithelial cells to regenerate properly.

Treatment of corneal LSCD is a trim challenge in ophthalmology [2-4]. Different diseases (such as aniridia, chemical, or thermal injury, ocular cicatricial pemphigoid, or Stevens Johnson syndrome), contact lens wear, antimetabolites, massive keratitis, as well as multiple surgeries can cause LSCD.

Patients with this condition are poor candidates for conventional corneal transplantation because grafting of the central cornea does not restore limbal cells [5]. Limbal autografts cannot always be performed, and limbal allografts carry potential graft-rejection disadvantages. In addition, the long-term outcome of the reconstructed epithelial surface is plagued with persistent epithelial defects, inflammation, and higher risk of secondary infection, which in turn can lead to revascularization and opacity of the corneal tissue. This pathological process promotes corneal instability, causing serious discomfort and morbidity to the patient [6].

Advances in corneal limbal epithelial cell culture have enabled researchers to develop new treatments [7]. The use of autologous grafts of functional epithelial corneal cell layers in humans was first described by Pellegrini et al. [8] in 1997, and since then several clinical protocols have started to test this new therapy [9-14]. This technique has some advantages over the classic limbal transplant, such as reduced postoperative inflammation [15]. Although some patients have been treated with this innovative therapy and have shown favorable results, more animal and clinical studies are still required to establish a treatment of proved efficacy for patients with LSCD [16-19].

The situation is completely different in patients with bilateral corneal stem cell deficiency where there is no healthy limbus to obtain the cells from. The therapeutic options for these patients include the use of allogenic limbal epithelial cells or an alternative source of healthy autologous epithelial cells that can mimic limbal epithelial cells. For the first option, living related donors or cadaveric corneas are required [20,21].

The use of supporting materials for transportation and transplantation of cultured epithelial cell sheets onto patients has improved the reproducibility of the technique. Materials most widely used include amniotic membrane [22-24] and fibrin glue [25]. The former presents potential benefits for epithelial cell adhesion, proliferation, and differentiation, while fibrin may be more convenient for interactions between the epithelium and stromal cells after surgery [26].

Surgical tissue adhesives have been developed to promote hemostasis and tissue sealing during surgery. Polymerizing fibrinogen with thrombin and calcium forms fibrin glue. When mixed together, these agents mimic the last stages of the clotting cascade to form a fibrin clot. Fibrinogen can be obtained from pooled, single-donor, or autologous blood and is usually isolated by the process of cryoprecipitation. The tensile strength and adhesive properties of the glue are proportional to the concentration of fibrinogen [27]. The thrombin component is generally derived from commercial bovine sources, which represents a risk for disease transmission.

It is worth mentioning the recent use of platelet poor plasma (PPP) as a cell supporter in corneal limbal epithelial cell transplantation [16]. We have been testing the use of plasma components with the aim of developing new techniques in the setting of experimental corneal surgery [28]. PPP can be clotted with the use of CaCl_2_. Clots can be easily handled in surgery and can simplify the transport of cell sheets. Once implanted on the surgical site, absorption of the PPP is rapidly achieved during wound healing. Its biocompatible and biodegradable properties prevent it from inducing foreign body reactions or extensive fibrosis [29]. PPP has no adhesive properties and, for this reason, the cell sheet has to be attached to the cornea with the aid of sutures or tissue glue.

In this study we intend to show the usefulness of epithelial corneal cell sheets mounted on a PPP support for allograft transplantation of rabbits with total LSCD. We also aim to prove its efficacy at 1 year postoperatively.

## Methods

### Animals

Thirty-one New Zealand White rabbits (CNEA, Argentina) weighing approximately 3,000 g each were used. Ten male rabbits acted as limbal donors. A sample of 13 ml of blood was drawn under strict aseptic conditions using a 21 G needle from each of the 21 female rabbits. A syringe preloaded with 1.3 ml of Anticoagulant Citrate Dextrose solution was used to avoid coagulation. A 15 min centrifugation at 72× g at 4 ºC was carried out in order to separate the plasma from the red and white cells; the plasma was then centrifuged for another 5 min at 1,000× g to obtain 7 ml of PPP in the upper part of the tube. PPP was stored in a sterile tube at –20 °C. The rabbits were treated in accordance with the guidelines of the Association for Research in Vision and Ophthalmology, Inc.: Statement for the Use of Animals in Ophthalmic and Vision Research [30].

### Biopsy, culture, and expansion of cells

A 2.5-mm^2^ biopsy of corneal limbal epithelial cells was obtained from the superior temporal part of the limbus of 10 male rabbit eyes, using a 1.8-mm-diameter trephine and a delicate forceps. This was preceded by gentle debridement of the conjunctiva and rinsing of the zone with saline to diminish cell contamination in the culture. The sample was immediately placed in a tube with Ham’s F12 containing 100 U/ml penicillin, 100 μg/ml streptomycin (Penicillin-Streptomycin, Gibco, Grand Island, NY), 2.5 μg/ml amphotericin B (Sigma-Aldrich, St. Louis, Mo), and 20 μg/ml gentamicin (Gibco) and transported to the laboratory.

The sample was washed three times in antibiotic-antimicotic solutions. A fourth wash removed the undesirable antimicrobials, and a fifth wash was used to perform sterility tests. The sample was enzymatically processed with trypsin (Invitrogen, Carlsbad, Ca) to obtain isolated cells. The rabbit limbal epithelial cells were seeded on a feeder layer of irradiated murine 3T3 fibroblasts and cultured in growing medium composed of a 3:1 mixture of Dulbecco's modified Eagle's Medium and Ham´s F12 (Hyclone, Logan, UT) supplemented with 10% fetal bovine serum, (Internegocios, Argentina), 0.4 μg/ml hydrocortisone (Sigma-Aldrich), 5 μg/ml transferrin (Gibco, Grand Island, NY), 5 μg/ml insulin (Sigma-Aldrich, Germany), 10^-10^ M cholera toxin A (Gibco, Carlsbad, CA), 1.8 x10^-4^ M adenine (Sigma-Aldrich), 2 x10^-11^ M triiodothyronine (Sigma-Aldrich), 100 U/ml penicillin, and 100 μg/ml streptomycin and placed in a 37 °C, 5% CO_2_ incubator. Once several colonies were grown, the feeder layer was detached from the culture flask, single cell suspensions were obtained from each culture, and cells were counted and seeded again or cryopreserved in the presence of 10% dimethyl sulfoxide (Sigma-Aldrich) and 20% fetal bovine serum (Internegocios, Argentina) in a density of approximately 10^6^ cells/ml.

### Graft assembling

Autologous PPP (extracted from the blood from rabbits with LSCD) was activated with CaCl**_2_**, which inhibits the blood-thinning effect of acid citrate dextrose solution. PPP was clotted in two circular culture chambers. Approximately 4×10^4^ male rabbit limbal epithelial cells/cm^2^ were seeded on a fibroblast feeder layer grown on each PPP clot, and cells formed a monolayer after a few days. To achieve cell stratification, epidermal growth factor (Invitrogen, Carlsbad, CA) was added to the culture medium to a final concentration of 10 ng/ml 4 days before surgery. In all cases, culture media were supplemented with 50,000 Kallikrein inhibitory units/ml aprotinin (Teva-Tuteur, Argentina) to prevent fibrinolysis. At the time of surgery all grafts contained a stratified cell sheet and could be easily detached from the chambers with forceps. Dehydration of the grafts during surgery was avoided with the application of hydroxypropyl methylcellulose drops every 5 min. Otherwise, serious damage to the grafted cells would ensue.

### Surgery

All surgical procedures were done under general anesthesia using a combination of intramuscular 1 mg/kg midazolam (Roche, Basel, Switzerland) and 70 mg/kg ketamine (Fada, Buenos Aires, Argentina) and topical anesthesia using sub-Tenon’s capsule injection of 1 ml of 4% lidocaine and proparacaine drops.

**Figure 1:**
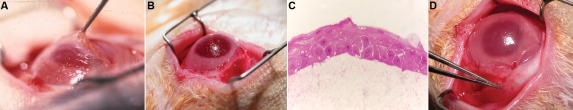
Allograft of corneal epithelial cell sheet on autologous platelet poor plasma clot. **A**, **B**, **D**: Once the lamellar keratectomy is performed, the rabbit stromal surface is covered with a corneal epithelial cell sheet, which is sutured with eight noncontinuous 10.0 nylon sutures. **C**: The corneal epithelial cell sheet presents a stratified epithelium consisting of four layers, inferior cells are polygonal and disclose a prominent nucleolus while cells of mid epithelium show irregular morphology, and superficial cells are flattened with a compact ovoid nucleus of homogeneous chromatin.

LSCD was induced in 21 female rabbits by mechanical deep anterior keratectomy, including the limbus (Figure 1A,B). Fifteen female animals received corneal epithelial cell sheet grafts that covered the entire cornea and were sutured with nylon 10.0 to the sclera. These sheets replaced one anterior third of the removed stroma and fit over the limbal margins. Post-surgical treatment included topical 0.3% ciprofloxacin (Bausch & Lomb, Buenos Aires, Argentina) given four times daily for 2 weeks and prednisolone 10 mg/ml (Allergan, Irvine, TX) given four times daily for 30 days. Clinical follow-up was performed for up to 1 year.

The control group was divided into two subgroups according to the post-anterior keratectomy treatment received. Control group 1 consisted of three animals that received no treatment after keratectomy and were sacrificed at 3 months. Control group 2 was formed by three animals, which were sutured with PPP clots lacking stem cell sheets and were followed for 1 year.

### Histopathology and immunofluorescence

Animals were sacrificed for corneal ablation. Five treated female rabbits and control group 1 were sacrificed at 90 days and the rest of the animals at 1 year. Corneal samples were cut at 10 μm thickness in a cryostat and processed for histopathological examination (hematoxilin and eosin staining) as well as for immunofluorescence studies to identify the expression of keratins in the corneal epithelium. Grafts were studied in the same way. mAb AE5 against keratins 3 and 12 (Chemicon, Temecula, CA) and mAb E6 against keratin 19 (Chemicon) were used. Briefly, sections were incubated overnight in a humid chamber at 4 °C with primary antibody diluted in PBS containing 0.2% (w/v) bovine serum albumin, 0.03% (v/v) Triton X-100, and 0.1% (w/v) sodium azide. Dilutions used were 1:300 for mAb anti K3/12 and 1:600 for mAb anti K19. After rinsing with PBS, sections were incubated for 60 min at 20 °C with biotinylated goat antimouse IgG (Vector, Burlingame, CA) followed by fluorescein-labeled antibiotin (Vector).

### Gender analysis

In month 4 of the follow-up period, two female rabbits were randomly selected to obtain a limbal biopsy. In month 7, another two rabbits were biopsied as well. A 2-mm diameter trephine, a blade number 11, and a delicate forceps were used to perform a 4 mm^2^ superficial flap of corneal epithelium in the superior corneal periphery.  Each sample was transported to the laboratory, and cells were expanded by single-cell suspension culture as described above and cryopreserved at −196 °C. Classic cytogenetic studies were carried out according to established protocols. Briefly, cells were thawed, seeded at approximately 3.10^3^ cells/cm^2^, and cultured until semiconfluence. To obtain metaphases, the following steps were followed: 2 h exposure to colchicine, feeder layer detachment, hypotonic shock in 0.075 M KCl, and 3:1 methanol–acetic acid fixation. Metaphases were karyotyped using G banding. G bands were obtained using Wright stain as follows: after 2 weeks at room temperature in dark containers, slides were pretreated for 20 s in 2× standard saline citrate solution (SSC) at 65 °C, washed with distilled water, and covered with a 1:2 Wright stain–Sorensen buffer for 2.5 min. Slides were quickly washed with abundant tap water and left in the dark until microscopic observation. G-banded slides were photographed at 1000× with an Olympus photomicroscope registering each metaphase’s landmarks. A minimum of 40 metaphase cells was examined for each individual.

## Results

### Treated group

A good stratified epithelium of four layers was achieved in all grafts by the time of surgery (Figure 1C). Autologous PPP clots showed good elasticity and transparency, facilitating the follow-up of cultures and the surgical technique. The grafts as well as the clots perfectly tolerated eight noncontinuous 10.0 nylon sutures (Figure 1D). Dehydration of PPP could be prevented during surgery. The right side up position of epithelial cell sheet grafts was clearly identified, and no rollover was experienced. There were no complications during the surgical procedure, with the exception of a small corneal perforation in one animal that evolved satisfactorily, achieving a clear cornea at the end of the protocol.

As expected, minimal signs of postoperative keratitis were seen until the second week. Of the 15 treated female rabbits, five were sacrificed at 90 days and eight at 1 year for histopathology and immunofluorescence studies. Of the remaining rabbits, one died of no apparent cause at 1 month and the other had to be sacrificed at 1 month because it progressed to microbial keratitis in the first weeks, did not respond to antibiotic therapy, and ended with a severe corneal infection and endophthalmitis.

**Figure 2:**
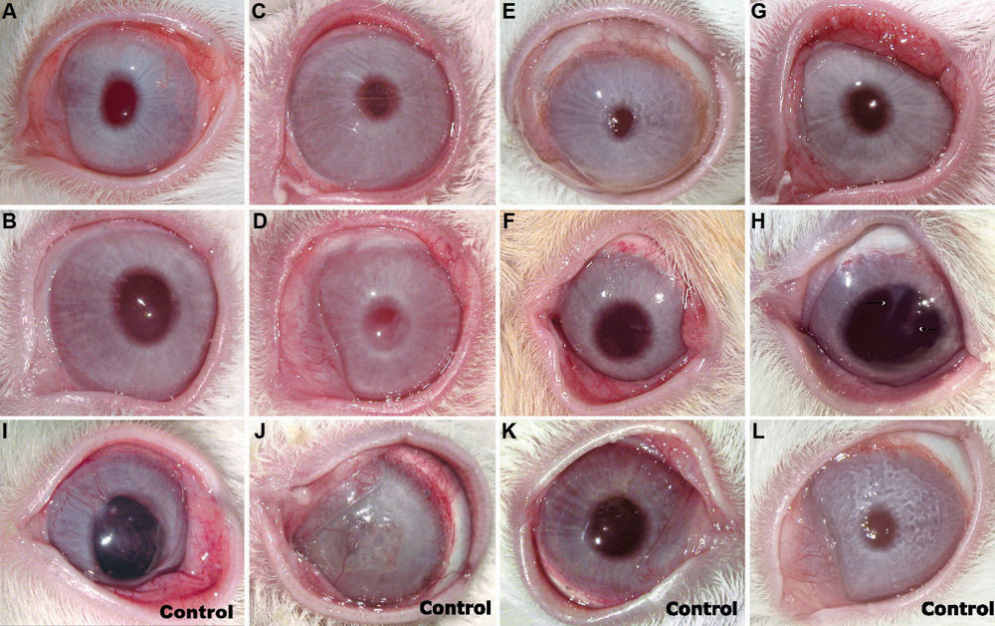
Clinical results at 1 year after surgery. Five eyes of the treated group displayed avascular and re-epithelialized corneas, with no signs of limbal stem cell deficiency (**B**, **C**, **E**, **F,** and **G**), one animal showed corneal neovascularization (**A**), one stayed with a stromal leucoma (**D**) and the other suffered from neovascularization that regressed thereafter leaving a stromal leucoma (**H**). Figure **I** shows one eye of the control group with no implant, at 3 months. The 3 animals of the control group implanted with PPP showed signs of limbal stem cell deficiency: one rabbit had to be sacrificed at 120 days because of a severe infection due to a IV grade corneal abscess (**J**), another spontaneously died at 240 days showing a small inferior leucoma and epithelial defects (**K**) and the remaining rabbit displayed total irregularity of the corneal surface at one year after surgery (**L**).

**Figure 3:**
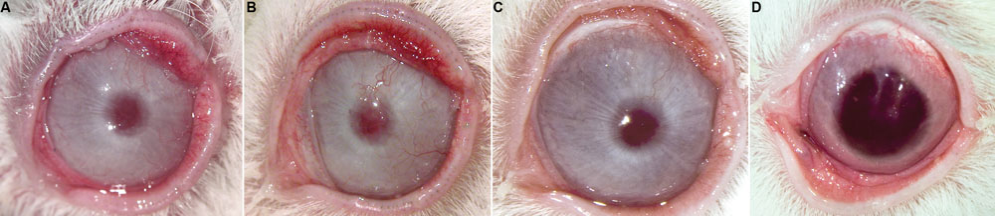
Neovascularization regression in one treated animal. One treated animal developed neovascularization of one-quarter of the cornea at 4 months after surgery (**A**) that began to regress thereafter without treatment to show no signs of neovessels at 12 months of follow-up (**D**). **B** and **C** show corneal appearance at 8 and 10 months, respectively.

At 1 year, only one animal out of eight showed a clinical sign of LSCD (one clock hour neovessel; Figure 2A), one presented a mild stromal leucoma in one quadrant (Figure 2D), and one developed neovascularization of one-quarter of the cornea at 4 months post surgery that began to regress thereafter without treatment to show no signs of neovessels at 12 months of follow-up (Figure 3). The remaining five rabbits showed a normal corneal surface, without epithelial defects (Figure 2B,C,E–G). Corneal clarity evolved toward improvement over time. The pupillary margin and the iris crypts could be identified in all animals. Corneal rejection, as described by Maumenee [31], was not seen.

**Figure 4:**
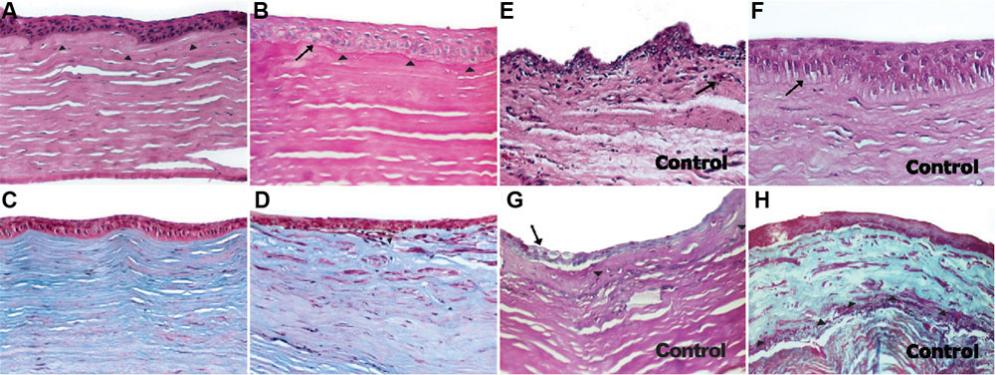
Histological results at 1 year. Histological corneal sections of treated animals (**A**–**D**) show an implanted corneal epithelium of a multilayer configuration with maturation from polygonal basal cells to flat superficial cells. Beneath the epithelium there is an acellular colagenous hyaline layer (arrows) with isolated keratocytes. The median and deep stroma as well as the Descemet membrane and endothelium have a normal appearance (hematoxylin and eosin stain; **A**). Periodic acid-Schiff  stain shows epithelial-like cells with clear cytoplasm at the basal layer of the corneal epithelium with a continuous basement membrane. A slightly periodic acid-Schiff positive acellular layer is seen between the epithelium and the normal corneal stroma (arrows; **B**). At the central area of the cornea, besides a thin homogenous layer beneath the epithelium, the remaining corneal stroma disclosed a normal lamellar configuration with interspread keratocytes (Masson stain; **C**). In the peripheral cornea, a thick layer of proliferated myofibroblasts and colagenous disposition deposits containing a few inflammatory cells is present between the implanted epithelium and the remaining corneal stroma (Masson stain; **D**). In the control group (**E**–**H)** an irregular regenerative epithelium from the limbal conjunctiva can be distinguished over the remaining stroma and a subepithelial scarring with inflammatory cells and neovascularization is present (Hematoxylin and eosin stain; **E**). A taller epithelium compared to normal/treated corneas is evident, with incomplete surface cell differentiation (Hematoxylin and eosin stain; **F**). Periodic acid-Schiff of the peripheral cornea discloses the presence of goblet cells within the corneal epithelium and a scarring of the underlying corneal stroma (**G**). Central cornea of a control eye shows a multilayered squamous-like corneal epithelium and a thick layer of active scar containing myofibroblasts, colagenous deposits, and inflammatory cells at the interface within the remaining corneal stroma (Masson stain; **H**).

At 90 days and at 1 year, histological corneal sections revealed a multilayered configuration with maturation from polygonal basal cells to flat superficial cells. Beneath the epithelium, an acellular colagenous hyaline layer with isolated keratocytes could be observed. The median and deep stroma as well as the Descemet membrane and endothelium showed a normal appearance (Figure 4A). Epithelial-like cells with clear cytoplasm at the basal layer of the corneal epithelium were seen, and a slightly Periodic Acid Schiff-positive acellular layer compatible with the basement membrane could be observed (Figure 4B). There were neither signs of vascular pannus nor avascular intraepithelial mucin cells on the implanted corneas. At the central area of the cornea, besides a thin homogenous layer beneath the epithelium, the remaining corneal stroma disclosed a normal lamellar configuration with interspread keratocytes (Figure 4C). In the peripheral cornea, a thick layer of proliferated myofibroblasts and colagenous disposition deposits containing a few inflammatory cells was present between the epithelium and the remaining corneal stroma (Figure 4D).

**Figure 5:**
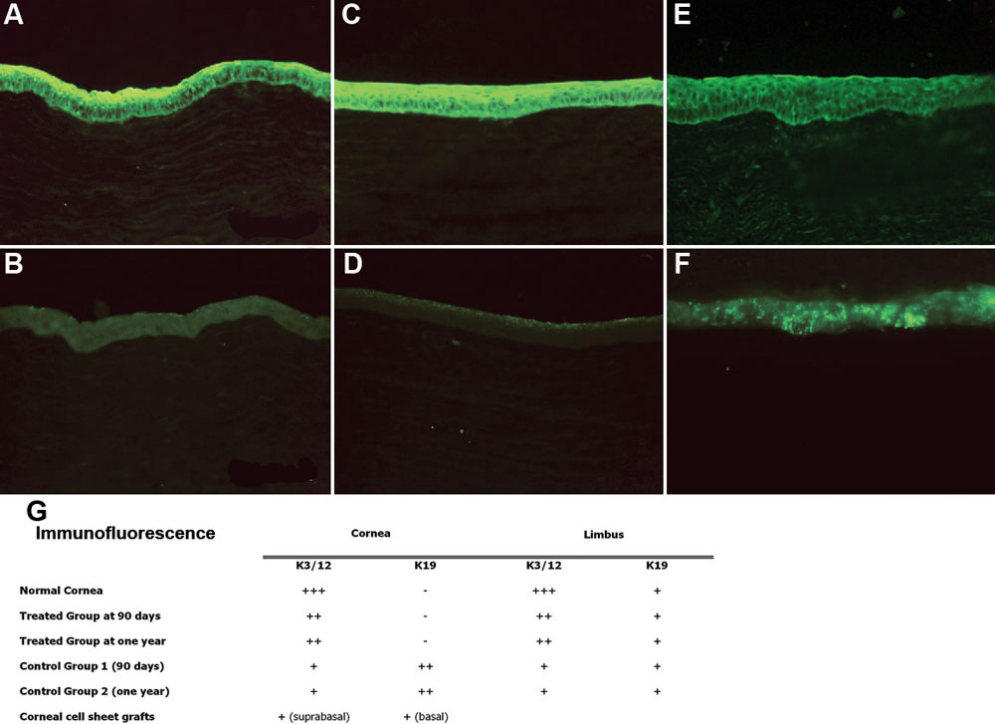
Immunofluorescence at 1 year. A normal corneal epithelium presents K3/12 (**A**) expression but no expression of K19 (**B**). Corneas of treated eyes also express K3/12 (**C**) but not K19 (**D**). In corneas of control 2 K3 is slightly expressed (**E**) and K19 is expressed in a patchy and erratic pattern (**F**). Table **G** discloses immunofluorescence results for central and peripheral corneas of normal, treated, and control eyes. Treated eyes show a similar expression than a normal eye, with strong K3/12 expression along the entire cornea, no expression of K19 in central cornea and a slight mark for K19 in the periphery. Both control groups express K19 along the central and peripheral cornea. Cultured epithelial sheet grafts show basal expression of K19 and suprabasal-layer expression of K3/12.

Immunofluorescence results showed expression of K3/12 and no expression of K19 (Figure 5C,D), similar to the pattern found in a normal corneal epithelium (Figure 5A,B). Expression of keratins on cultured epithelial sheet grafts showed basal expression of K19 and suprabasal-layer expression of K3/12 (Figure 5G). Allogenic donor chromosomes could be detected in one of the two rabbits biopsied at each time (4 and 7 months after surgery).

### Control group C1 (no sheet, no platelet poor plasma, 90 days)

All animals showed limbal ischemia and persistent epithelial defects (defined as epithelial defects for more than 2 weeks after initial epithelialization). They suffered from irregular corneal surface with moderate stromal ulcers. Two rabbits developed neovascularization after 75 days. Visualization of the pupillary margin was fairly blurry (Figure 2I). Histological sections revealed a thin regenerative and erratic epithelium with minimal surface cell differentiation and pronounced fibroblastic proliferation (data not shown). Basement membrane could not be distinguished. An elevated expression of K19 and weak expression of K3/12 were found (data not shown).

### Control group C2 (no sheet, platelet poor plasma clot, 1 year)

One animal died at 240 days of no apparent cause, and another had to be sacrificed at 120 days because of a severe infection due to a IV grade corneal abscess [32].

All rabbits developed limbal ischemia at 90 days and showed persistent epithelial defects and an irregular corneal surface with multiple stromal ulcers, which caused a blurry vision of the pupillary margin, and almost no distinguishable iris crypts at the same time. These animals showed LSCD signs with corneal keratitis, epithelial irregularities, and vascularization of the center and periphery of the cornea. At 1year total irregularity of the corneal surface was seen (Figure 2L).

The rabbit that spontaneously died at 240 days showed a small inferior leucoma and epithelial defects in two clockwise hours (Figure 2K). At the time of sacrifice, the rabbit with the corneal abscess presented permanent 360^o^ corneal neovascularization and a large central opacity that hindered visualization of the pupil (Figure 2J).

Histological sections revealed an irregular regenerative epithelium from the limbal conjunctiva over the remaining stroma and a subepithelial scarring with inflammatory cells and neovascularization (Figure 4E). A taller epithelium compared to normal/treated corneas was evident, with incomplete surface cell differentiation (Figure 4F). Intraepithelial mucin cells could be identified in one animal at 120 days (Figure 4G). All animals showed a multilayered squamous-like corneal epithelium and a thick layer of active scar containing myofibroblasts, colagenous deposits, and inflammatory cells at the interface within the remaining corneal stroma (Figure 4H). indirect immunofluorescence revealed slight K3/12 expression (Figure 5E). K19 was expressed in a patchy pattern (Figure 5F).

## Discussion

Our results demonstrate that full thickness allografts of bioengineered limbal epithelium mounted on an autologous PPP support can be an alternative treatment for corneal allograft transplantation. The implanted cell sheets favored re-epithelialization and survived for a long period of time without the use of systemic chronic immune suppression. The promising results observed at 90 days post surgery could still be confirmed at 1 year post surgery.

To our knowledge, this is the first report on the use of autologous plasma and calcium as support for homologous epithelial cell sheets. This method provides not only an economic advantage over commercially available fibrin glues but also a safer matrix with respect to disease transmission and immunogenicity. In vitro allogenic epithelial cells could be satisfactorily grown over PPP clots.

Autologous PPP clots are easily handled, which facilitates the grafting of limbal corneal epithelial cell sheets. No difficulties were encountered with the suture technique since the graft was elastic and strong enough to tolerate eight noncontinuous 10.0 nylon sutures. Autologous PPP is a cost-effective option and its obtainment and the cell cultures have been carried out following standard procedures. The transparency of the PPP clots simplified follow-up of cell sheet cultures.

Disease or destruction of the corneoscleral limbus leads to ingress of conjunctiva-derived cells, including goblet cells, and blood vessels onto the normally avascular corneal surface, affecting its optical properties and leading to visual impairment or blindness [33]. Immune privilege of the eye has long been known as the mechanism by which the majority of orthotropic corneal allografts are permanently accepted [34]. It is believed that this immune privilege is actively acquired and maintained by immune regulatory forces in the eye. These forces include the avascular nature of the cornea and the presence of local inhibitory factors in corneal epithelial and endothelial cells [35]. Since we grafted male allograft cells over a denudated female corneal and limbal stroma, we expected to observe minimal signs of graft rejection, but none of the treated animals showed this complication. The corneal epithelium was found to be clinically and immunohistologically normal, evidencing nonpathological re-epithelialization. No signs of immunological reaction were seen during follow-up after allogenic transplantation, including epithelial rejection. This unwanted condition, defined as an epithelial line (Khodadoust line) or diffuse punctate corneal epitheliopathy plus diffuse conjunctival inflammation is fairly frequent in lamellar keratoplasty [36]. A possible explanation for this desirable outcome is that graft rejection occurs in a chronic way, allowing the graft to work as a support for the regeneration of part of the host stem cell population. This estimation is in line with the observations of Daya et al. [37] who investigated the outcome of an *ex vivo* expanded stem cell allograft as a treatment for LSCD in 10 patients. They analyzed donor cell survival in seven patients, using PCR, and could prove it only in two patients but not beyond 9 months postoperatively. The methodology used by us to detect the presence of donor DNA has inherent limitations in sensitivity but not in accuracy; this means that the presence of the Y chromosome undoubtedly indicates that there are donor cells still present in the cornea, but no conclusion can be made based on the absence of the Y chromosome. Indeed, the percentage of XY metaphases determined in each sample depends on the proliferative capacity of the cultured cells and the isolation procedure and has no relation to the percentage of XY cells present in the epithelium. At the same time, a negative result does not prove the absence of donor cells in the biopsy. We consider very interesting the fact that donor cells forming part of the epithelium of treated eyes could be found at 7 months postoperatively, especially when the biopsy was obtained from the corneal periphery where the probability to take autologous cells is augmented, because those donor cells that we could expand in vitro must have maintained an undifferentiated state to stay at the corneal periphery for such a long time. Demonstrating the persistence and acceptance of an XY genotype graft over an XX receptor is of paramount importance in the pursuit of a successful therapeutic strategy. Furthermore, these allogenic grafts might be useful for corneal gene delivery to treat some corneal epithelial dystrophies of genetic origin, such as Meesmann dystrophy [38].

Corneal neovascularization can lead to vision loss and it is often difficult to manage. Newly or already formed corneal neovessels elevate the risk of subsequent graft rejection after corneal transplantation [39]. Medical and surgical therapies used to reduce corneal neovessels include corticosteroids, nonsteroidal anti-inflammatory agents, laser photocoagulation, needle diathermy [40], and recently, the use of bevacizumab [41]. Many of these therapies have not only demonstrated limited success but also demonstrated associated adverse effects. As is seen in patients, once graft rejection occurs, graft failure ensues, and a second keratoplasty and limbal graft is required to restore the ocular surface. In our study, corneal neovascularization with spontaneous resolution only occurred in the treated group. With this favorable result, we remark on the usefulness of the implanted grafts to maintain the integrity and healthiness of the cornea.

In general, a favorable outcome—defined as clear cornea—could be detected at 30 days post surgery. In the treated group, corneal transparency remained unchanged up to 1 year of follow-up. Clear nonmucin-secreting cells were seen on the implanted animals as seen on lamellar corneal implanted humans, probably as a result of constant re-epithelization, which  may represent transient amplifying cells. In contrast, in the control groups, corneal neovascularization, surface irregularities, and opacity started to be visible from week 3 until the end of the protocol. Based on these findings, we conclude that the persistence of the implanted tissue and the healing of the receptor limbus jointly avoid, in turn, corneal invasion of the conjunctiva and corneal neovascularization.

Keratins 19 and 3/12 have been chosen merely to compare the expression of normal treated and control corneal epithelium. At present, there are no consistent data that can relate clinical success to a determined expression pattern. To characterize the putative corneal stem cells, the presence of morphologic features and several markers are used in combination with the absence of cornea-specific differentiation markers, but the identification of limbal stem cells is still under debate [42].

The above results and the keratin immunofluorescence outcomes suggest that cultivated limbal epithelial transplantation is a useful approach to the treatment of LSCD, restoring a feasible microenvironment in the ocular surface and securing a corneal epithelial phenotype.

In conclusion, the allograft corneal epithelium was able to restore and maintain a healthy eye surface appearance without signs of graft rejection. The reported technique seems to be a promising procedure for the treatment of bilateral ocular surface diseases and constitutes an important step toward the development of a new therapeutic strategy in patients. The access to corneas from eye banks is a difficult and expensive option, and people with bilateral LSCD would benefit from allografts of epithelial corneal sheets cultured on PPP clots as support.
